# Melatonin prescribing practices and the provision of sleep hygiene/parent-led sleep behavioural Interventions in S-CAMHS, Aneurin Bevan University Health Board (ABUHB)- Service evaluation as part of quality improvement project

**DOI:** 10.1192/bjo.2021.920

**Published:** 2021-06-18

**Authors:** Kathryn Speedy, Lokesh Nukalapati, Kathryn Speedy, Megan Davies-Kabir

**Affiliations:** Aneurin Bevan University Health Board

## Abstract

**Aims:**

To identify the number of patients currently on melatonin

To determine the average duration of use of melatonin in patients under the care of S-CAMHS in ABUHB

To investigate whether behaviour interventions were tried and reinforced from time to time

To identify any areas of improvement

**Method:**

Data were collected at St. Cadoc's hospital, in January, 2021. S-CAMHS database was used. Out of total 346 patient currently being managed with pharmacological therapies, 115 (33.2%) are currently on melatonin. 57/115 were randomly selected as a sample for this this project. Patient notes and EPEX software were also used to collect information regarding the sleep management practices.

**Result:**

During analysis, it was noticed that within the sample, only 46 patients were actively on melatonin. Melatonin is prescribed for sleep related issues in ASD (8/46), ADHD (15/46), ASD and ADHD (10/46), ADHD and mood disorder (0/46), ASD and mood disorder (6/46), ADHD and behaviour difficulties (2/46), ASD with behaviour difficulties (1/46), mood disorder (4/46).

39/46 patients are currently on melatonin for more than a year (85%). These patients also include 10 patients who have been using melatonin for 5 years or more.

35 patients (76%) reported improved sleep or some benefit from melatonin.

Evidence for implementation of parent-led sleep behavioural interventions:

Prior to commencing melatonin- Clear evidence available for 35 patients only (76%). These interventions were however not deemed helpful by most of the service users.

While prescribing melatonin- Clear evidence available for 39(85%) patients. Evidence base for melatonin was also discussed during this visit.

During last follow-up visit- Evidence available for 31 patients only (67%).

**Conclusion:**

Majority of patients under S-CAMHS ABUHB remain on melatonin therapy for longer than one year. Most of these patients have reported benefit from this therapy and preferred to remain on it despite being informed about evidence base for melatonin. Also, there is evidence for implementation of sleep behavioural interventions prior to prescribing melatonin, however their benefit remains unclear.

Recommendations:

The quality of education on sleep hygiene offered should be assessed and improved if needed

Formal group sessions/workshops on sleep hygiene/parent-led sleep behavioural interventions at regular intervals might be useful in reducing the chances of long term polypharmacy or unlicensed drugs

Use of outcome measures such as Child Sleep Habits Questionnaire at intervals can be helpful in identifying any improvement from educational/pharmacological interventions

S-CAMHS database (for patients actively on medications) needs a review and update

